# Post-silking Factor Consequences for N Efficiency Changes Over 38 Years of Commercial Maize Hybrids

**DOI:** 10.3389/fpls.2017.01737

**Published:** 2017-10-11

**Authors:** Keru Chen, Tony J. Vyn

**Affiliations:** Agronomy Department, Purdue University, West Lafayette, IN, United States

**Keywords:** maize hybrids, Post-silking dry matter accumulation, post-silking N accumulation, Partial factor productivity, nitrogen internal efficiency

## Abstract

Hybrid selection in maize (*Zea mays* L.) over the decades has increased post-silking dry matter (PostDM) and nitrogen (PostN) accumulation, often with an accompanying increase in one or more N use efficiency (NUE) metrics such as partial factor productivity (PFP), N conversion efficiency (NCE), and N internal efficiency (NIE). More certainty on the underlying mechanisms of how PostDM and PostN changes over time have contributed to NUE gains or losses in modern-era hybrids can only be realized by directly comparing hybrids of different eras in the context of production-system-relevant management systems. A two-year and two-location field study was conducted in Indiana with two N rates (55 and 220 kg N ha^−1^), three plant densities (54,000, 79,000, and 104,000 plants ha^−1^) and eight commercial hybrids that were released by a single seed company from 1967 to 2005. The main treatment effects of N rate, density, and hybrid dominated the PostDM and PostN responses, and there were no significant two-way or three-way interactions. Total dry matter at maturity gains averaged 80 kg ha^−1^ year^−1^ of hybrid release when averaged over locations, plant densities and N rates. Total N contents at maturity increased 0.68 kg ha^−1^ year^−1^, primarily due to annual increases in grain N content (0.8 kg ha^−1^ year^−1^). Post-silking N uptake rate increased 0.44 kg ha^−1^ year^−1^ for these era hybrids in more favorable production site-years. Slopes of grain N concentration increases per unit PostN gain were similar for all hybrids. Gains in average PFP over time were considerably higher at the low N rate (0.9 kg ha^−1^ year^−1^) than at the high N rate (0.3 kg kg^−1^ year^−1^). Hybrid gains in NIE were evident from 1967 to 1994, but not thereafter. The low N rate and higher plant densities also increased relative NIE and NCE values, but without hybrid interactions. There was no consistent trend of NIE or NCE gains in these hybrids primarily because grain and whole-plant N concentrations didn't decline over the decades at either N rate, and because NIE and NCE were often plant-density dependent.

## Introduction

Maize hybrid selection over time has resulted in large whole-plant and grain yield gains along with higher whole-plant plant N uptake on an area basis. Until recently, there has been less clarity about N efficiency changes over time despite a strong societal and industry interest in applying less N fertilizer per unit of production while limiting reactive N losses to the environment. Discovery of how possible N efficiency improvements have contributed to maize grain yield changes is an ongoing question worthy of intense research involving integrated genetic and management system variables. However, the multiple definitions of N use efficiency have further hindered scientific certainty about efficiency gains or losses over time, and their underlying mechanisms, in maize and other cereal grains.

Thirty-two years ago, Anderson et al. (1985) found that higher N internal efficiency (NIE = Grain weight/Total N uptake at maturity) in prolific maize hybrids, regardless of N rates, was due to their higher N remobilization during grain filling compared to semi-prolific hybrids. They also suggested that hybrid selection under low N conditions could be more effective in selecting hybrids that combined high nitrogen use efficiency (NUE) and high yields. Subsequently, many studies have focused on changes in maize N uptake dynamics over time (Muchow, 1994; Pan et al., 1995; Coque and Gallais, 2007; Bender et al., 2013; Chen et al., 2015). Many of these studies discovered higher post-silking N uptake (PostN) in more recent hybrid eras. For example, Ciampitti and Vyn (2013) concluded in their review of past studies involving measurement of whole-plant N at maturity that 56% of grain N was derived from PostN in hybrids from 1991 to 2011 vs. just 50% for hybrids released before 1991. Other recent studies also found that the higher yield in current hybrids was associated with greater PostN (Worku et al., 2007; Cirilo et al., 2009).

Many agronomic indices have been used in describing NUE in maize production, including partial factor productivity (PFP), agronomic efficiency (AE_N_), nitrogen internal efficiency (NIE), apparent crop recovery efficiency (RE_N_), and physiological efficiency (PE_N_) (Dobermann, 2005, 2007; Fixen et al., 2015). Partial factor productivity, which is the ratio of yield vs. N applied, can be easily recorded in either on-farm studies or research station trials (Dobermann, 2005, 2007; Ladha et al., 2005). Doberman and Cassman (2002) reported PFP increased from 41 kg grain kg^−1^ N applied in 1980 to 58 kg grain kg^−1^ N applied in 2000 in the US when the average N fertilizer rate was 145 kg N ha^−1^. Ciampitti and Vyn's (2014) review paper reported that PFP averaged 57 kg grain kg^−1^ N across time intervals from 1880 to 1990 as well as from 1991 to 2012 in US field research experiments. According to the data from USDA National Agricultural Statistics Service, PFP at the farm level was further increased to 61 kg grain kg^−1^ N across selected states in 2010 and even greater in 2014 (66 kg grain kg^−1^ N; USDA, 2016a,b). Ciampitti and Vyn (2012) observed an average NIE (kg grain kg^−1^ N uptake) increase from 49.7 kg kg^−1^ in old era hybrids (1940–1990) to 56 kg kg^−1^ in new era hybrids (1991–2011) in their review of disparate maize research studies from around the world.

The NIE metric can be understood in two ways. The first approach is to treat NIE as a product of nitrogen conversion efficiency [NCE, the ratio of total dry matter at maturity (TDM_R6_) to total N content at maturity (TNU_R6_)] and harvest index (HI) (Ciampitti and Vyn, 2012; Gastal et al., 2015; Mueller and Vyn, 2016). Nitrogen conversion efficiency is also the inverse of plant N concentration at maturity (PNC_R6_). In this way, NIE was explained by both efficiency of converting N into dry matter and the ability of transferring dry matter to grain (Gastal et al., 2015). Mueller and Vyn (2016) found that NCE levels were significantly higher in New Era (1991–2014) hybrids than in Old Era (1903–1990) hybrids at both silking and at maturity. Mueller and Vyn (2016) also found that NCE at maturity explained 51% of variance in NIE, whereas HI only explained 24%, when these hybrid eras were combined.

The second approach is to treat NIE as a ratio of nitrogen harvest index (NHI) and grain N concentration (Sadras, 2006; Ciampitti and Vyn, 2012). Sadras (2006) found that grain N concentration (GNC) explained more variation of NIE than NHI for all cereals, legumes, and oilseeds he examined. Ciampitti and Vyn (2012) found that GNC of old hybrids (1940–1990) explained 46% of NIE variation, while GNC explained 65% of NIE variation in newer hybrids (1991–2011). The dilution of PNC_R6_ and GNC contributed to higher NIE in more recent hybrids (Cassman et al., 2002; Ciampitti and Vyn, 2012). However, the large decline in PNC_R6_ and GNC [~10% from Old Era 1940–1990 to New Era 1991–2011, as observed by Ciampitti and Vyn, 2012, also negatively affects protein concentration in maize grain (Cassman et al., 2002)].

Moll et al. (1982) documented that NIE differences among hybrids under low N was mainly due to variation in utilization of accumulated N, whereas hybrid variation at the high N rate was due to their ability to uptake applied N more proficiently. Anderson et al. (1985) reported that the higher grain yield under a high N rate did not lead to a higher NIE. Modern maize hybrids may or may not result in higher N recovery; for example, a higher N rate directly affected N uptake by increasing PostN in older as well as more recent maize hybrids (Chen et al., 2015).

Quantification of the benefits of higher N rates to improve plant N status can be realized by the nitrogen nutrition index (NNI), a ratio of actual N concentration to critical N content for obtaining maximum biomass (Lemaire and Gastal, 1997). Gallais and Coque (2005) showed that NNI at silking correlated with leaf senescence at 3 weeks after silking. However, hybrid era changes in NNI at varied N levels, and its correlation with NUE, has not been well documented in the maize literature.

Plant density's impact on maize NUE has also been reported very infrequently, especially in comparisons of newer vs. older hybrids, despite the huge importance of plant density as a management variable. In one such report, Chen et al. (2015) observed that higher densities increased leaf N content (but not N concentration) at silking and increased leaf N remobilization during grain filling period, which had negative impacts on PostN. Ciampitti and Vyn (2011) reported that higher plant density increased NUE by increasing both NIE and nitrogen recovery efficiency (NRE) with 165 and 330 kg N ha^−1^ applied to two relatively modern hybrids. Meanwhile, the rate of grain yield gain over time could be impacted by plant densities (Duvick, 2005; Chen et al., 2016). Hence, it is necessary, especially when considering hybrid genetic improvements over time, to evaluate how plant density could impact post-silking dynamics of DM and N and their relationship to NUE.

Clarification on the real hybrid era changes in N efficiency parameters, and the underlying mechanisms for those changes, can only be realized when hybrids from multiple eras are grown side-by-side in the same environments using relevant management systems. Therefore, the objectives in our study were to determine: (1) how dry matter and N dynamics from silking to maturity changed over 38 years of commercial hybrid development for different N rates and plant densities, (2) how NUE metrics changed under different N levels and plant densities, (3) the nature of the potential trade-off between grain yield and NUE at high N input conditions, and (4) how the variation of NIE changed via its components (i.e., grain dry matter and total N uptake at maturity, plant N concentration and HI, and grain N concentration and NHI) in the different treatment combinations.

## Materials and methods

### Experimental design and management

A field study was conducted at ACRE (Agronomy Center for Research and Education, 40°28′07″N, 87°00′25″W), West Lafayette, IN, USA and PPAC (Pinney Purdue Agricultural Center, 41°26′41″N, 86°56′41″W), Wanatah, IN, USA in 2013 and 2014. The soil was Chalmers silty-clay loam (Fine-silty, mixed, superactive, mesic Typic Endoaquolls) in 2013 and Raub-Brenton complex (Fine-silty, mixed, superactive, mesic Aquic Argiudolls) in 2014 at ACRE. The soil at PPAC was Sebewa loam (Fine-loamy over sandy or sandy-skeletal, mixed, superactive, mesic Typic Argiaquolls) in both years. Average soil pH, organic matter, Mehlich-3 P, and Mehlich-3 K were 6.9, 3.7 g 100 g^−1^, 22 mg kg^−1^, 106 mg kg^−1^ at ACRE in 2013; 6.7, 4.4 g 100 g^−1^, 17 mg kg^−1^, 92 mg kg^−1^ at PPAC in 2013; and 6.2, 2.9 g 100 g^−1^, 75 mg kg^−1^, 236 mg kg^−1^ at ACRE in 2014, 6.2, 4.8 g 100 g^−1^, 27 mg kg^−1^, 129 mg kg^−1^ at PPAC in 2014. Soil N was not measured at sowing in this study; however, it was measured at V14 and R1 stages at both ACRE (2013 and 2014) and PPAC (2013 only) in immediately adjacent maize hybrid studies planted on the same day in the same field where no N fertilizer was added (de Oliveira Silva et al., 2017). Soil ${\text{NH}}_{4}^{+}$ ranged from 3.3 to 4.8 mg kg^−1^ and ${\text{NO}}_{3}^{-}$ ranged from 1.8 to 3.5 mg kg^−1^ to a 30-cm depth at these locations in spring before N application (de Oliveira Silva et al., 2017). In both years, maize was planted after soybean at ACRE, and after maize at PPAC. The 2013 ACRE location was chisel plowed in the fall and field cultivated in the spring. The 2014 ACRE field site was strip-tilled in both fall and spring with a Soil Warrior® (Environmental Tillage Systems Inc.) using coulter-based soil engaging tools. At PPAC, the tillage system was chisel plow in the fall and field cultivation in the spring for PPAC in both years.

Treatments were arranged in a split-split plot design in both years at both locations. Nitrogen rate was the main plot - 55 kg N ha^−1^ (55N) or 220 kg N ha^−1^ (220N). Plant density was the sub-plot – 54,000, 79,000, and 104,000 plants ha^−1^. Hybrid was the sub-sub-plot, including 8 commercial DeKalb hybrids released from 1967 to 2005. They were: 2005, DKC61-69 (VT3); 2005, DKC61-72 (RR2); 2003, RX752 (VT3); 2003, RX752RR2 (RR2); 1994, RX730 (Conventional); 1982, DK636 (Conventional); 1975, XL72AA (Conventional), and 1967, XL45 (Conventional). VT3 hybrids are resistant to European corn borer, corn rootworm, and glyphosate. RR2 hybrids are resistant to glyphosate. Conventional hybrids have no transgenic pest-resistant and glyphosate resistant traits. These eight commercial hybrids were selected in consultation with Dekalb maize breeders because they were widely grown in the US Corn Belt following their release and had similar relative maturity days ranging from 111 to 115 days.

Six blocks were planted at ACRE and three blocks were planted at PPAC. All plots were 10 m long and 3.04 m wide with 4 rows and 0.76 m row spacing. Planting dates were 14 May, 2013 and 25 April, 2014 at ACRE and 1 Jun, 2013 and 5 May, 2014 at PPAC. Nitrogen was side-dressed as urea-ammonium nitrate (UAN, 28% N) applied 30 days after planting (DAP) in 2013 and 33 DAP in 2014 at ACRE and 38 DAP in 2013 and 24 DAP in 2014 at PPAC. All UAN was injected in mid-row positions with a DMI Nutri-Placer 2800. All grass and broadleaf weeds in the plot areas were controlled with a combination of pre-emerge residual herbicides as well as a single post-emerge application at approximately the V5 stage. All maize seeds were treated in a similar manner with Acceleron™ (Difenoconazole, Fludioxonil, Mefenoxam, and Thiamethoxam). Force 3G (Tefluthrin) was soil-applied at planting with all maize hybrids to control corn rootworm.

Weather data for ACRE were collected from Purdue University-Indiana State Climate Office at station “ACRE-West Lafayette” (http://www.iclimate.org/), and for PPAC were collected from station “Wanatah 2 WNW, IN US” (http://www.ncdc.noaa.gov/cdo-web). Weather recording began with the planting dates at each site-year and continued until biomass harvest at maturity on 24 September, 2013 and 15 September, 2014 at ACRE, and on 22 October, 2013 and 29 September, 2014 at PPAC.

### Biomass harvest

At ACRE, R1 biomass harvest was taken at 7 days (2013) and 0 days (2014) after 50% silking (average of all hybrids). At PPAC, R1 biomass harvest was taken at 2 days (2013) and 4 days (2014) after 50% silking (average of all hybrids). More precise timing of R1 harvest on the actual mean dates of 50% silk emergence (following daily flowering measurements in each plot) could not be achieved because of weather and labor resource constraints. R6 biomass harvest was completed after all treatments reached black layer (representative ears of each hybrid from multiple replications were sampled to insure all treatments had reached black layer). For all biomass harvests, the sampling area was 3.04 m^2^ for each plot. All plants in the sampling area were cut at soil level and weighed to determine the total fresh weight. Five representative plants were then chosen as subsamples from each plot. For the R1 harvest, subsamples were separated into leaf, stem (with husk) and earshoot in both years. For the R6 harvest, subsamples were separated into leaf, stem (with husk), grain and cob for three blocks in ACRE and PPAC in both years. The other three blocks in ACRE were separated into stover (stems, leaves, and husks) and ears (grain and cob) at ACRE in both years. Fresh weights were taken for all samples. All subsamples were dried at 60 °C at ACRE for 5–7 days until they reached a stable dry weight. All subsamples from the first three blocks were weighed, ground and sent to A&L Great Lakes Lab (Fort Wayne, Indiana) for determination of plant N composition using combustion analysis (AOAC International 990.03, 1995).

### Equations

Post-silking dry matter accumulation was calculated by the following formulas:

$$\begin{array}{l} \text{Post}-\text{silking dry matter accumulation }\left(\text{PostDM}\right)\\ \;=\text{ Total dry matter at maturity}\\ \;-\text{Total dry matter at silking} \end{array}$$

Dry matter remobilization from vegetative organs (leaf or stem) was determined as the dry matter lost between vegetative and reproductive stages by using the following formulas:

$$\begin{array}{l} \text{Leaf Remobilized DM }({\text{RemDM}}_{\text{leaf}})=\text{Leaf DM at silking}\\ \;-\text{Leaf DM at maturity}\\ \text{Stem Remobilized DM }({\text{RemDM}}_{\text{stem}})=\text{Stem DM at silking}\\ \;-\text{Stem DM at maturity} \end{array}$$

Post-silking N uptake (PostN), remobilized N (RemN), leaf remobilized N (RemN_leaf_), stem remobilized N (RemN_stem_) and cob remobilized N (RemN_cob_) were determined as the N content lost between vegetative and reproductive stages utilizing the same equations that were employed in Chen et al. (2015).

Whole-plant N concentration at maturity was calculated as one of the explanatory variables for nitrogen internal efficiency:

$$\text{Whole }-\text{ plant N concentration at maturity }=\;\frac{(\text{Leaf N content}+\text{Stem N content}+\text{Cob N content}+\text{Grain N content})\text{ at maturity}}{\left(\text{Leaf dry matter}+\text{Stem dry matter}+\text{Cob dry matter}+\text{Grain dry matter}\right)\text{ at maturity}}$$

Nitrogen use efficiency metrics included partial factor productivity, nitrogen internal efficiency, nitrogen conversion efficiency, and nitrogen nutrition index; these were determined by following formulae:

$$\begin{array}{l} \text{ Partial factor productivity }(\text{PFP})\;=\;\frac{\text{Grain Dry Matter at maturity}}{\text{N applied}}\\ \text{Nitrogen internal efficiency }\left(\text{NIE}\right)=\;\frac{\text{Grain Dry Matter at maturity }}{\text{Total N uptake at maturity}}=\;\frac{\text{Total dry matter at maturity}}{\text{Total N uptake at maturity}}\;\times \text{ harvest index}\\ \;=\frac{1}{\text{Whole}-\text{plant N concentration}}\times \text{ harvest index}\\ \;=\text{N conversion efficiency }\left(\text{NCE}\right)\;\times \text{ harvest index}\\ \text{Nitrogen internal efficiency }\left(\text{NIE}\right)=\;\frac{\text{Grain Dry Matter at maturity }}{\text{Total N uptake at maturity}}=\frac{\text{Grain Dry Matter at maturity}}{\text{Grain N uptake at maturity}}\times \text{ N harvest index}\\ \;=\frac{1}{\text{Grain N concentration}}\times \text{ N harvest index}=\;\frac{\text{N harvest index}}{\text{Grain N concentration}}\\ \text{ N conversion efficiency }\left(\text{NCE}\right)\;=\;\frac{\text{Total dry matter at maturity}}{\text{Total N uptake at maturity}}\\ \text{ Nitrogen nutrition index }\left(\text{NNI}\right)\;=\;\frac{\text{Actual N concentration at silking}}{\text{Critical N concentration at silking}}=\frac{\text{Actual N concentration at silking}}{3.4\;\times \text{ Total dry matter at }{\text{silking}}^{\left(-0.37\right)}} \end{array}$$

The formula for critical N concentration at silking can be found in Gastal et al. (2015), and the NNI parameters for maize were defined in Plénet and Lemaire (1999).

### Statistical analysis

Statistical analysis was conducted in SAS 9.4 (SAS Institute Inc., 2013). Nitrogen, density and hybrids were fixed effects. Year and block (nested in each year) were treated as random effects. Only the first three blocks (1–3) were used in statistical analysis due to lack of N test data for the last three blocks (4–6). Locations were analyzed separately. Three error terms for this split-split-plot design was main plot error: E(a) – Block(year) × Nitrogen, sub-plot error: E(b) – Pooled Block(year) × Density and Block(year) × Density × Nitrogen and sub-sub-plot error: E(c) – Pooled Block(year) × Hybrid, Block(year) × Hybrid × Nitrogen, Block(year) × Hybrid × Density, and Block(year) × Hybrid × Nitrogen × Density. If the random terms had P(F > F0) less than 0.05 for most of the variables, then the random term(s) was pooled with the corresponding error terms. Hence, Year × Nitrogen was pooled with E(a), Year × Nitrogen × Density and Year × Density was pooled with E(b), Year × Hybrid, Year × Nitrogen × Hybrid, Year × Density × Hybrid, and Year × Nitrogen × Density × Hybrid was pooled with E(c). The final model was used in SAS:

$$\begin{array}{l} y=\mu +\alpha_{i}+\beta_{j}+\alpha \beta_{ij}+\gamma_{k}+\alpha \gamma_{ik}+\beta \gamma_{jk}+\alpha \beta \gamma_{ijk}+\tau_{l}\\ \;\;\;\;\;\;\;\;+\delta_{m}(l)+\alpha \delta_{im_{\left(l\right)}}+\alpha \beta \delta_{ijm_{\left(l\right)}}+\alpha \beta \gamma \delta_{ijkm}{}_{{}_{\left(l\right)}}+\epsilon_{ijkml} \end{array}$$

where, μ is the grand mean, α is N rate effect (*i* = 1, 2), β is density effect (*j* = 1, 2, 3), γ is hybrids effect (*k* = 1, 2, …, 8), αβ is N rate and density interaction, αγ is N rate and hybrid interaction, βγ is density and hybrid interaction, α*βγ* is N rate, density and hybrid three-way interaction, τ is year effect (*l* = 1, 2), δ is block effect that nested in each year (*m* = 1, 2, 3), αδ is N rate and block interaction effect, α*βδ* is N rate, density and block interaction effect, α*βγδ* is N rate, density, hybrid and block interaction, and ε is the error term.

Linear correlations were conducted by using “Proc Reg” in SAS. Log-transformation was used on the linear correlations between NIE and grain dry matter at maturity, NIE and total N content at maturity, NIE and whole-plant N concentration at maturity, NIE and HI, NIE and NHI, as well as between NIE and grain N concentration at maturity. The R^2^ of fitted linear model was used as the percentage of NIE that was explained by each explanatory variable. Annual rates of increase were calculated using the slope of linear fit based on the mean values of each parameter of interest for each hybrid. Bilinear function was conducted in SPSS 23.0 (IBM Corp., Armonk, N.Y., USA). The equation used for bilinear function was *Y* = *a*_1_ + *b* × *X at X* < *x*_0_, *and Y* = *a*_2_ *at X* ≥ *x*_0_. Parameter estimation is based on the loss function, which is (*y* − *ŷ*)^2^.

## Result

### Weather conditions

**Figure 1:**
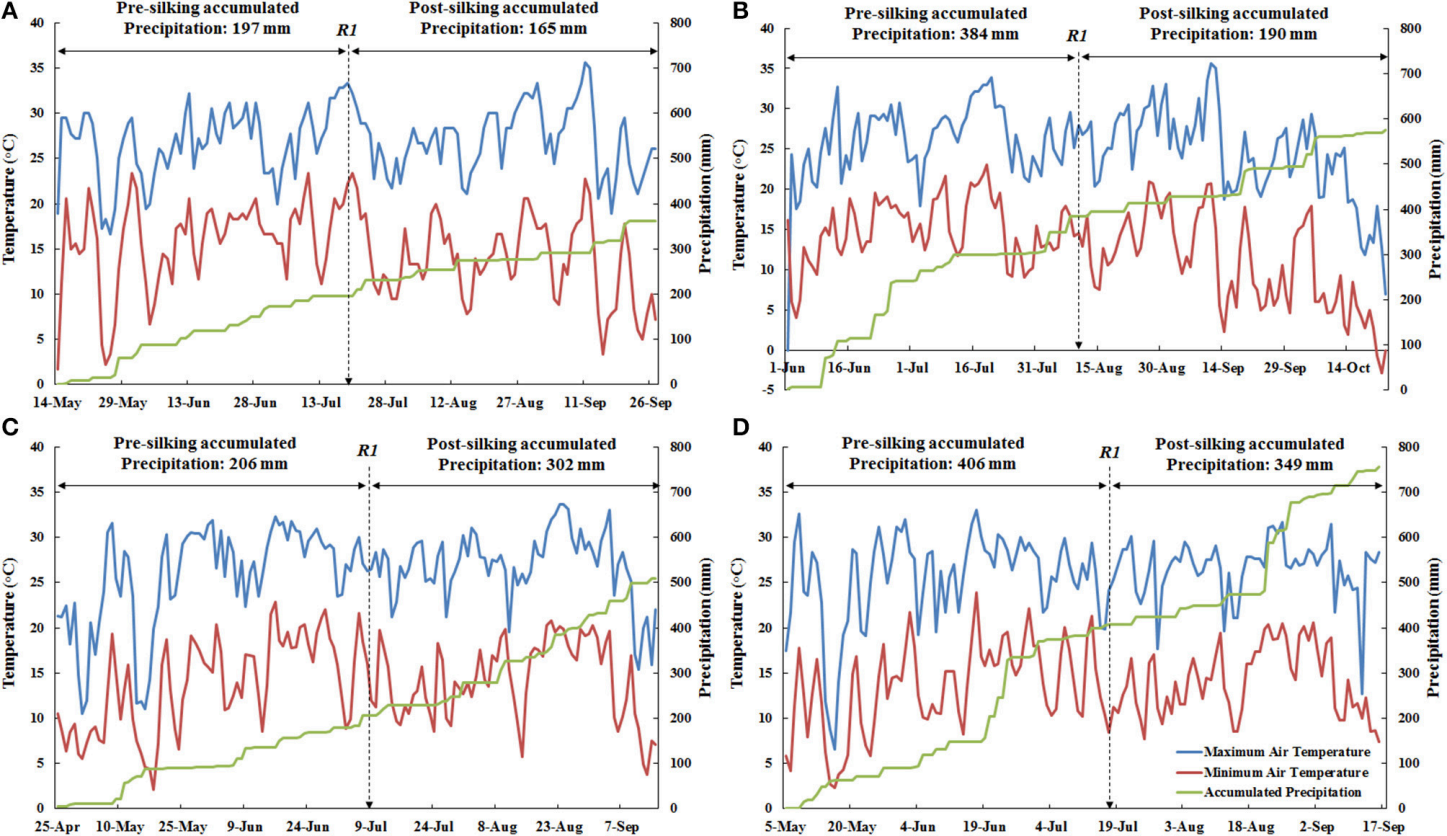
The weather conditions for the two sites in both 2013 and 2014, including maximum air temperature (°C, blue line), minimum air temperature (°C, red line), and accumulated precipitation (mm, green line). For each environment, maximum air temperature and minimum air temperature shared the primary y-axis (left) and accumulated precipitation used secondary y-axis (right). Date for actual 50% silking (R1) was marked for each environment by a dashed arrow. The amount of pre-silking and post-silking accumulated precipitation are also marked in each environment. **(A)**, Represents ACRE, 2013; **(B)**, Represents PPAC, 2013; **(C)**, Represents ACRE, 2014; and **(D)**, Represents PPAC, 2014.

There was less accumulated precipitation at ACRE than PPAC in the pre-silking period as well as the post-silking period during both years (Figure 1). For 2013, the accumulated precipitation was 187 mm less at ACRE during the pre-silking period than PPAC, and 25 mm less during the post-silking period (Figures 1A,B). For 2014, PPAC had 200 mm higher accumulated precipitation than ACRE during the pre-silking period and 47 mm more during the post-silking period (Figures 1C,D). In general, 2014 had 164 mm higher accumulated precipitation than 2013 across locations (Figure 1). The cumulative pre-silking precipitation was similar in ACRE between 2013 and 2014 (only 9 mm higher in 2014), which was also the case for PPAC (only 22 mm higher in 2014). However, the larger cumulative precipitation difference between years for ACRE and PPAC was during the post-silking period, which was 137 mm higher in 2014 for ACRE (Figures 1A,C) and 159 mm higher in 2014 for PPAC (Figures 1B,D). In contrast, the temperature ranges were similar between the four environments, except for the occurrence of minimum temperatures below 0°C after planting and right before harvest at PPAC in 2013 (Figure 1).

### Dry matter changes from silking to maturity

Although the 1967 hybrid always had the lowest dry matter at silking, total dry matter at silking did not increase as era increased from 1975 to 2005 at either location (Tables 1, 2). An N rate and hybrid interaction was observed for total dry matter at silking (TDM_R1_) at PPAC (Table 2). This interaction was due to a higher TDM_R1_ response to increased N rate for 2005 hybrids, which increased 1.1 and 0.6 Mg ha^−1^ for 2005VT3 and 2005RR2, respectively, compared to a relatively small TDM_R1_ increase of −0.1 to 0.4 Mg ha^−1^ for 1967 to 2003 hybrids (Table 2).

**Table 1:** ANOVA of main effects for total dry matter at silking, and grain, total dry matter at maturity, and post-silking dry matter accumulation, proportion of post-silking dry matter in total dry matter at maturity at ACRE.

		**Dry matter at silking**	**Dry matter at maturity**		**Post-silking**	
		**Total**	**Grain**	**Total**	**PostDM**	**PostDM / TDM_*R*6_**
		**Mg ha^−1^**	**Mg ha**^**−1**^		**Mg ha^−1^**	**%**
N rate	55N	9.5	10.7	20.5	11	53
(N)	220N	9.7	12.2	22.5	12.8	57
	LSD	0.5	1.1	1.7	1.4	3
Density	54,000	8.8	11.1	20.9	12.1	58
(D)	79,000	9.7	11.7	21.8	12.1	55
	104,000	10.2	11.4	21.8	11.5	53
	LSD	0.4	0.3	0.6	0.6	2
Hybrid	1967	7.9	9.1	17.4	9.5	54
(H)	1975	9.8	11	21.6	11.8	54
	1982	9.9	10.9	21.9	12	55
	1994	9.6	11.4	20.9	11.3	54
	2003RR2	9.8	12	21.9	12.1	55
	2003VT3	9.7	12.1	21.8	12.1	55
	2005RR2	10.1	12.4	23.3	13.2	56
	2005VT3	9.9	12.6	23.1	13.3	57
	LSD	0.4	0.5	0.9	0.9	3
*F*-test	N	ns	0.015	0.031	0.022	0.044
	D	<0.001	0.001	0.003	ns	<0.001
	H	<0.001	<0.001	<0.001	<0.001	ns
	N × D	ns	ns	ns	ns	ns
	N × H	ns	ns	ns	ns	ns
	D × H	ns	ns	ns	ns	ns
	N × D × H	ns	ns	ns	ns	ns

**Table 2:** ANOVA of main effects for total dry matter at silking, and grain, total dry matter at maturity, and post-silking dry matter accumulation, proportion of post-silking dry matter in total dry matter at maturity at PPAC.

		**Dry matter at silking**		**Dry matter at maturity**			**Post-silking**		
		**Total**		**Grain**		**Total**	**PostDM**	**PostDM / TDM_*R*6_**
		**Mg ha**^**−1**^		**Mg ha**^**−1**^			**Mg ha^−1^**	**%**
N rate	55N	8.7		7.4		15.2	6.5	42
(N)	220N	9		9.5		18.2	9.2	50
	LSD	0.5		1		1.6	1.6	6
Density	54,000	8.3		8.6		16.6	8.3	49
(D)	79,000	9		8.6		17	8	46
	104,000	9.4		8.1		16.5	7.1	42
	LSD	0.3		0.3		0.6	0.5	2
		**55N**	**220N**	**55N**	**220N**			
Hybrid	1967	8.1	8.0	6.3	7.8	13.9	5.8	41
(H)	1975	8.9	9.3	6.6	8.9	16.4	7.3	44
	1982	8.8	9.0	7.2	8.8	17.1	8.2	48
	1994	8.7	8.8	7.6	9.3	16.3	7.6	46
	2003RR2	8.8	9.2	7.5	9.7	16.8	7.8	45
	2003VT3	9.1	9.0	7.9	10.4	17.6	8.5	47
	2005RR2	8.7	9.3	7.9	10.1	17.4	8.4	48
	2005VT3	8.6	9.7	8.1	11.0	18.1	9	49
	LSD	0.3		0.4		0.7	0.8	3
*F*-test	N	ns		0.003		0.005	0.008	0.023
	D	<0.001		0.013		ns	<0.001	<0.001
	H	<0.001		<0.001		<0.001	<0.001	<0.001
	N × D	ns		ns		ns	ns	ns
	N × H	0.011		0.023		ns	ns	ns
	D × H	ns		ns		ns	ns	ns
	N × D × H	ns		ns		ns	ns	ns

*Significant N rate and hybrid interactions are presented for each N level*.

There were considerably more dry matter differences among hybrids at maturity than at silking. The total dry matter at maturity (TDM_R6_) of 2005 hybrids were 6% higher than that of 2003 hybrids at ACRE (Table 1) but the same as 2003 hybrids at PPAC (Table 2), while the 2005 hybrids were 13 and 11% higher than the average of hybrids prior to 2000s at ACRE and PPAC, respectively. The yearly increasing rate for TDM_R6_ was 92 kg ha^−1^ year^−1^ in ACRE and 67 kg ha^−1^ year^−1^ in PPAC. The higher TDM_R6_ in 2000s hybrids was mostly due to higher grain dry matter at maturity (GDM) at both locations (Tables 1, 2). The N rate and hybrid interaction for GDM at PPAC (Table 2) was due to the greater gain in GDM of 2005VT3 at 220N, which increased 36% (2.9 Mg ha^−1^) compared to an average gain of 28% (2.0 Mg ha^−1^) for the remaining hybrids. There was no consistency of hybrid era effects on leaf or stem DM remobilization at either location (Tables S1, S2).

Hybrid and management impacts on PostDM gains were readily apparent. The two 2005 hybrids had 10% higher PostDM compared to the average of 2003 hybrids at ACRE, but were the same as 2003 hybrids at PPAC (Tables 1, 2); they were also 19 and 20% higher than the average of hybrids prior to 2000s at ACRE and PPAC, respectively (Tables 1, 2). There was no era effect on the ratio of PostDM to TDM_R6_ at either location. The higher N rate increased both PostDM (by 1.8 and 2.7 Mg ha^−1^) and the proportion of PostDM in TDM_R6_ (by 4 and 8% at ACRE and PPAC, respectively (Tables 1, 2). In contrast, increased plant density reduced the proportion of PostDM in TDM_R6._

### Plant component N concentration and content changes at maturity

Both vegetative organs and grain had large variations in N concentrations among hybrids at maturity, but there was no consistent era impact. The 1967 hybrid always had the highest grain N concentration (GNC) and whole-plant N concentration (PNC_R6_) at both locations (Tables 3, 4). The 2003 hybrids had 10–12% lower GNC and PNC_R6_ compared with the 1967 hybrid, but the 2005 hybrids' GNC and PNC_R6_ was within a range of just 5–8% lower.

**Table 3:** ANOVA of main effects for leaf, stem, cob, grain, and whole-plant N concentrations at maturity at ACRE.

		**N concentration at maturity**						
		**Leaf**	**Stem**			**Cob**	**Grain**	**Whole-plant**
		**g 100 g**^**−1**^						
N rate	55N	0.87	0.38			0.31	1.05	0.79
(N)	220N	1.23	0.5			0.33	1.22	0.98
	LSD	0.12	0.05			0.04	0.06	0.06
Density	54,000	1.14	0.46			0.29	1.22	0.94
(D)	79,000	1.04	0.43			0.32	1.09	0.86
	104,000	0.97	0.43			0.35	1.08	0.85
	LSD	0.05	0.02			0.02	0.04	0.03
			**Low-D**	**Med-D**	**High-D**			
Hybrid	1967	1.11	0.57	0.51	0.57	0.32	1.24	0.97
(H)	1975	0.99	0.40	0.44	0.44	0.31	1.12	0.85
	1982	1.01	0.40	0.42	0.38	0.33	1.14	0.86
	1994	1.14	0.48	0.47	0.46	0.40	1.04	0.86
	2003RR2	1.08	0.45	0.39	0.40	0.30	1.09	0.86
	2003VT3	1.07	0.42	0.42	0.40	0.28	1.09	0.87
	2005RR2	1.01	0.44	0.39	0.37	0.32	1.17	0.89
	2005VT3	0.98	0.48	0.40	0.40	0.30	1.18	0.91
	LSD	0.05	0.03			0.03	0.03	0.02
*F*-test	N	<0.001	0.001			ns	0.044	0.006
	D	<0.001	0.013			<0.001	<0.001	<0.001
	H	<0.001	<0.001			<0.001	<0.001	<0.001
	N × D	ns	ns			ns	ns	ns
	N × H	ns	ns			ns	ns	ns
	D × H	ns	0.007			ns	ns	ns
	N × D × H	ns	ns			ns	ns	ns

*When density by hybrid interaction is significant, hybrid means are presented for each density level*.

**Table 4:** ANOVA of main effects for leaf, stem, cob, grain, and whole-plant N concentrations at maturity at PPAC.

		**N concentration at maturity**								
		**Leaf**		**Stem**		**Cob**		**Grain**		**Whole-plant**
		**g 100 g**^**−1**^								
N rate	55N	0.75		0.36		0.33		0.91		0.69
(N)	220N	1.07		0.45		0.33		1.1		0.87
	LSD	0.08		0.07		0.04		0.08		0.07
Density	54,000	0.98		0.39		0.28		1.05		0.81
(D)	79,000	0.92		0.41		0.33		1		0.78
	104,000	0.83		0.41		0.38		0.97		0.75
	LSD	0.05		0.02		0.03		0.02		0.01
		**55N**	**220N**	**55N**	**220N**	**55N**	**220N**	**55N**	**220N**	
Hybrid	1967	0.8	1.09	0.37	0.44	0.29	0.31	0.88	1.07	0.85
(H)	1975	0.73	1.08	0.35	0.42	0.41	0.46	0.93	1.12	0.76
	1982	0.77	1.05	0.42	0.58	0.3	0.27	0.93	1.13	0.75
	1994	0.8	1.2	0.34	0.37	0.32	0.39	0.93	1.13	0.78
	2003RR2	0.7	1.07	0.33	0.41	0.32	0.33	0.89	1.07	0.76
	2003VT3	0.78	1.08	0.34	0.45	0.32	0.28	0.89	1.14	0.77
	2005RR2	0.7	0.97	0.36	0.44	0.33	0.31	0.86	1.04	0.77
	2005VT3	0.72	0.99	0.37	0.49	0.34	0.3	1.01	1.18	0.79
	LSD	0.07		0.05		0.05		0.06		0.02
*F*-test	N	<0.001		0.016		ns		0.003		0.001
	D	<0.001		ns		<0.001		<0.001		<0.001
	H	<0.001		<0.001		<0.001		<0.001		<0.001
	N × D	ns		0.039		ns		ns		ns
	N × H	0.009		<0.001		0.001		0.043		ns
	D × H	ns		ns		ns		ns		ns
	N × D × H	ns		ns		ns		ns		ns

*Significant N rate and hybrid interactions are presented for each N level*.

Nitrogen rate and hybrid interactions were observed for leaf N concentration at maturity (LNC_R6_), stem N concentration at maturity (SNC_R6_), cob N concentration at maturity (CNC_R6_) and GNC at PPAC (Table 4). In general, the 1994 hybrid had the largest response to N rate with gains of 51% in LNC_R6_, 23% in CNC_R6_, and 28% in GNC. The 1982 hybrid had the largest response to N rate in SNC_R6_ with mean gains from 0.42 to 0.58 g 100 g^−1^. In contrast, 2005VT3 hybrid experienced little change with the higher N rate in leaf, cob and grain N concentrations at maturity.

In general, increasing density decreased organ N concentrations at both locations (Tables 3, 4). At maturity, a hybrid and density interaction was observed for stem N at ACRE (Table 3), where the two 2005 hybrids had much reduced stem N from low to medium density (from 0.46 to 0.38 g 100 g^−1^), while the 1967 hybrid had almost zero reduction (from 0.57 to 0.56 g 100 g^−1^).

Hybrid era influences on total N uptake at maturity (TNU_R6_) were consistent at both locations (Tables 5, 6). The annual rate of increase in TNU_R6_ was 0.70 and 0.66 kg ha^−1^ year^−1^ at ACRE and PPAC, respectively. The era effect on TNU_R6_ was primarily due to the increase in grain N content (GNU) in more recent hybrids since leaf N content (LNU_R6_), stem N content (SNU_R6_), and cob N content (CNU_R6_) at maturity did not show any consistent changes over the decades (Tables 5, 6). The annual rate of increase for GNU was 0.66 and 0.42 kg ha^−1^ year^−1^ at ACRE and PPAC, respectively. The N rate by hybrid interaction of GNU and TNU_R6_ at PPAC were due to 9 kg ha^−1^ higher GNU and 6 kg ha^−1^ higher TNU_R6_ responses of 2000s hybrids to higher N rate than was observed in earlier hybrids.

**Table 5:** ANOVA of main effects for leaf, stem, ear, and total N content at silking, leaf, stem, cob, grain and total N content at maturity, leaf, stem, cob and total remobilized N, post-silking N uptake, proportion of post-silking N uptake in grain N content at maturity (PostN/GNU), and proportion of post-silking N uptake in total N content at maturity (PostN/TNU_*R*6_) at ACRE.

		**N content at silking**				**N content at maturity**					**Remobilization**				**Post-silking**		
		**Leaf**	**Stem**	**Ear**	**Total**	**Leaf**	**Stem**	**Cob**	**Grain**	**Total**	**Leaf**	**Stem**	**Cob**	**Total**	**PostN**	**PostN/GNU**	**PostN/TNU_*R*6_**
		**kg ha**^**−1**^				**kg ha**^**−1**^					**kg ha**^**−1**^				**kg ha^−1^**	**%**	**%**
N rate	55N	69	43	8.5	120	26	20	4.7	113	164	42	23	3.8	69	44	37	25
(N)	220N	81	63	8.5	152	38	27	5.5	148	219	43	36	2.9	82	67	44	30
	LSD	7	11	1.0	17	5	3	1.0	16	23	7	8	1.1	10	10	6	4
Density	54,000	68	52	8.7	128	32	24	4.7	138	199	36	28	3.9	68	70	49	34
(D)	79,000	76	53	9.1	138	32	23	5.1	130	190	43	30	4.0	78	52	39	26
	104,000	81	53	7.8	141	33	23	5.6	124	185	48	30	2.2	80	44	33	22
	LSD	4	4	1.0	6	2	2	0.4	5	8	4	4	1.1	7	10	6	4
Hybrid	1967	60	49	11.0	120	28	23	5.6	114	170	32	26	6.0	64	50	42	28
(H)	1975	77	58	4.1	139	32	25	5.2	124	186	45	33	-1.1	77	47	37	25
	1982	76	53	4.7	134	35	24	5.2	126	190	41	29	-0.5	70	56	43	28
	1994	73	50	9.9	132	32	25	5.9	120	183	41	25	4.1	70	50	39	26
	2003RR2	79	50	8.9	137	31	22	5.0	132	190	48	28	3.9	80	53	38	26
	2003VT3	77	49	9.2	135	31	22	4.5	133	190	46	27	4.7	78	55	38	27
	2005RR2	78	57	10.3	146	36	23	5.4	147	212	42	34	5.0	81	66	42	29
	2005VT3	79	56	9.8	145	33	23	5.0	150	211	45	33	4.8	83	67	43	31
	LSD	4	3	1.3	6	2	2	0.5	7	9	4	4	1.4	7	10	7	4
*F*-test	N	0.006	0.005	ns	0.005	0.002	0.003	ns	0.002	0.002	ns	0.009	ns	0.037	0.002	0.032	0.044
	D	<0.001	ns	0.029	<0.001	ns	ns	0.002	<0.001	0.006	<0.001	ns	0.003	0.003	<0.001	<0.001	<0.001
	H	<0.001	<0.001	<0.001	<0.001	<0.001	0.001	<0.001	<0.001	<0.001	<0.001	<0.001	<0.001	<0.001	<0.001	ns	0.006
	N × D	ns	ns	ns	ns	ns	ns	ns	ns	ns	ns	ns	ns	ns	ns	ns	ns
	N × H	ns	0.001	ns	ns	0.046	ns	0.047	ns	ns	ns	ns	ns	ns	ns	ns	ns
	D × H	ns	ns	ns	ns	ns	ns	ns	ns	ns	ns	ns	ns	ns	ns	ns	ns
	N × D × H	ns	ns	ns	ns	ns	ns	ns	ns	ns	ns	ns	ns	ns	ns	ns	ns

**Table 6:** ANOVA of main effects for leaf, stem, ear, and total N content at silking, leaf, stem, cob, grain, and total N content at maturity, leaf, stem, cob and total remobilized N, post-silking N uptake, proportion of post-silking N uptake in grain N content at maturity (PostN/GNU), and proportion of post-silking N uptake in total N content at maturity (PostN/TNU_R6_) at PPAC.

		**N content at silking**				**N content at maturity**					**Remobilization**				**Post-silking**		
		**Leaf**	**Stem**	**Ear**	**Total**	**Leaf**	**Stem**	**Cob**	**Grain**	**Total**	**Leaf**	**Stem**	**Cob**	**Total**	**PostN**	**PostN/GNU**	**PostN/TNU_R6_**
		**kg ha**^**−1**^				**kg ha**^**−1**^					**kg ha**^**−1**^				**kg ha^−1^**	**%**	**%**
N rate	55N	48	31	5.2	85	18	15	4.0	68	104	30	17	1.3	48	19	25	23
(N)	220N	63	50	6.1	118	29	20	4.8	105	158	34	30	1.2	65	40	36	26
	LSD	6	10	1.0	16	5	3	0.7	16	23	4	8	0.9	11	7	9	3
Density	54,000	52	41	6.4	99	23	16	3.8	92	135	29	24	2.6	56	36	36	26
(D)	79,000	57	39	5.5	102	24	18	4.5	87	134	33	21	1.0	55	31	32	26
	104,000	57	42	5.1	103	23	17	4.8	80	125	34	24	0.2	58	22	23	22
	LSD	3	2	0.6	3	2	1	0.4	4	6	2	2	0.7	3	5	6	3
Hybrid	1967	47	39	10.2	96	18	18	5.0	78	119	29	21	5.2	56	23	24	21
(H)	1975	57	43	2.4	102	23	19	4.2	81	127	34	24	-1.8	55	25	27	26
	1982	56	40	2.3	99	26	20	4.3	79	129	30	21	-2.0	49	31	36	28
	1994	52	38	6.6	96	25	18	5.3	81	129	27	20	1.3	49	33	36	27
	2003RR2	58	41	6.3	105	23	17	4.2	86	129	36	24	2.1	62	24	24	20
	2003VT3	58	41	6.0	105	25	17	4.1	91	136	33	24	1.9	59	31	30	24
	2005RR2	58	40	5.5	103	23	15	4.2	93	136	34	24	1.3	60	33	33	25
	2005VT3	58	43	6.1	107	25	16	3.9	100	145	33	27	2.1	62	38	35	26
	LSD	4	2	0.9	5	2	1	0.4	5	7	3	3	0.9	5	8	9	4
*F*-test	N	0.002	0.005	ns	0.003	0.002	0.008	0.032	0.002	0.002	ns	0.009	ns	0.013	0.001	0.019	0.023
	D	0.001	ns	0.001	0.071	ns	0.007	<0.001	<0.001	0.002	<0.001	0.033	<0.001	ns	<0.001	<0.001	0.016
	H	<0.001	0.001	<0.001	<0.001	<0.001	<0.001	<0.001	<0.001	<0.001	<0.001	<0.001	<0.001	<0.001	0.002	0.014	0.002
	N × D	0.043	ns	ns	ns	ns	ns	ns	ns	ns	ns	ns	ns	ns	ns	ns	ns
	N × H	0.035	ns	ns	ns	ns	ns	<0.001	<0.001	0.017	ns	ns	ns	0.028	ns	ns	ns
	D × H	ns	ns	ns	ns	ns	ns	ns	ns	ns	ns	ns	ns	ns	ns	ns	ns
	N × D × H	ns	0.026	ns	ns	ns	ns	ns	ns	ns	ns	ns	ns	ns	ns	ns	ns

Increasing GNU over the hybrid eras was accomplished by gains in both PostN and RemN. The RemN was 10 and 9 kg ha^−1^ higher in 2000s hybrids than hybrids prior to 2000 at ACRE and PPAC, respectively. Additionally, the PostN was 11 and 4 kg ha^−1^ higher in 2000s hybrids than hybrids prior to 2000 at ACRE and PPAC, respectively (Tables 5, 6).

Leaves contributed most to RemN at both locations (Tables 5, 6). Leaves accounted for 57 and 58% of RemN, whereas stems contributed 39 and 40% of RemN at ACRE and PPAC, respectively. Increased N fertilizer rate increased RemN_stem_ but not RemN_leaf_ at both locations, whereas increasing density enhanced RemN_leaf_ but had no impact on RemN_stem_.

### N use efficiency changes over hybrid era

Partial factor productivity increased dramatically over time. The annual rate of increase in PFP was 1.0 and 0.8 kg kg^−1^ year^−1^ at 55N, and 0.4 and 0.3 kg kg^−1^ year^−1^ at 220N, at ACRE and PPAC, respectively (Table 7). The interaction of N rate by hybrids for PFP was due to the lower PFP reduction for the 1967 hybrid at ACRE (112 vs. 142 kg kg^−1^ for average of rest hybrids) and the lower reduction for 1967 and 1975 hybrids at PPAC (Table 7). There was no consistent era benefit to NIE; in fact, the 2003 and 1994 hybrids always tended to have from 3 to 6 kg kg^−1^ higher NIE compared with other hybrids at both locations and N rates. Hybrid differences in NCE demonstrated no era pattern and seemed to be mostly due to genetic variation, and NCE was directly affected by their whole-plant N concentration—which lead to a lower NCE for the 1967 hybrid at both N rates and both locations (Table 7).

**Table 7:** ANOVA of main effects for N use efficiency variables: PFP, partial factor productivity; NIE, nitrogen internal efficiency; NCE, N conversion efficiency; HI, harvest index; NHI, nitrogen harvest index; NNI, nitrogen nutrition index; at ACRE and PPAC.

		**ACRE**								**PPAC**								
		**PFP**		**NIE**	**NCE**	**HI**	**NHI**	**NNI**		**PFP**		**NIE**	**NCE**	**HI**	**NHI**		**NNI**	
		**kg kg**^**−1**^						**g g**^**−1**^		**kg kg**^**−1**^							**g g**^**−1**^	
N rate	55N	194		67	130	0.52	0.68	0.85		134		71	149	0.48	0.64		0.65	
(N)	220N	55		56	103	0.54	0.68	1.07		43		60	115	0.52	0.66		0.87	
	LSD	19		4.0	12.0	0.02	0.02	0.12		12		5.0	16.0	0.02	0.02		0.09	
Density	54,000	122		58	109	0.53	0.69	0.96		91		65	127	0.52	0.67		0.78	
(D)	79,000	129		63	119	0.53	0.68	0.97		89		66	132	0.50	0.65		0.76	
	104,000	124		63	121	0.52	0.67	0.96		86		67	137	0.49	0.63		0.75	
	LSD	5		2.5	5.5	0.01	0.02	0.06		4		2.4	6.8	0.01	0.01		0.07	
		**55N**	**220N**					**55N**	**220N**	**55N**	**220N**				**55N**	**220N**	**55N**	**220N**
Hybrid	1967	156	44	56	107	0.52	0.67	0.85	1.06	114	36	61	122	0.51	0.66	0.65	0.67	0.86
(H)	1975	193	52	61	120	0.51	0.66	0.84	1.09	120	41	63	136	0.47	0.61	0.65	0.62	0.88
	1982	186	53	59	120	0.50	0.66	0.81	1.05	130	40	63	136	0.47	0.61	0.61	0.63	0.85
	1994	190	56	64	119	0.54	0.66	0.83	1.04	139	42	68	132	0.52	0.63	0.63	0.62	0.83
	2003RR2	205	58	65	119	0.55	0.69	0.86	1.06	136	44	68	132	0.51	0.65	0.67	0.67	0.89
	2003VT3	202	59	65	118	0.55	0.70	0.87	1.02	144	47	69	134	0.52	0.65	0.67	0.66	0.90
	2005RR2	208	61	61	115	0.53	0.70	0.86	1.13	143	46	68	133	0.51	0.66	0.70	0.65	0.88
	2005VT3	212	62	61	113	0.54	0.71	0.88	1.13	147	50	68	131	0.52	0.67	0.70	0.68	0.89
	LSD	14		3.5	8.0	0.01	0.01	0.07		10		3.5	9.8	0.01	0.02		0.07	
*F-*test	N	<0.001		0.001	0.002	0.012	0.365	0.005		<0.001		0.002	0.003	0.006	0.038		0.002	
	D	0.030		<0.001	0.002	ns	0.021	ns		0.016		ns	0.023	0.001	<0.001		ns	
	H	<0.001		<0.001	<0.001	<0.001	<0.001	<0.001		<0.001		<0.001	<0.001	<0.001	<0.001		<0.001	
	N × D	ns		ns	ns	ns	ns	ns		ns		ns	0.032	ns	ns		ns	
	N × H	<0.001		ns	ns	ns	ns	0.005		<0.001		ns	ns	ns	0.019		ns	
	D × H	ns		ns	ns	ns	ns	ns		ns		ns	ns	ns	ns		ns	
	N × D × H	ns		ns	ns	ns	ns	ns		ns		ns	ns	ns	ns		ns	

*Significant N rate and hybrid interactions are presented for each N level. Nitrogen nutrition index is always presented at each N level*.

Harvest index (HI) and nitrogen harvest index (NHI) did not increase consistently over time. Harvest index plateaued after 1990s at both N rates and both locations, except for the 220N rate at ACRE, where HI plateaued with the 2003 hybrids followed by a slight decline with the 2005RR2 hybrid (Table 7). Harvest index was 6 and 8% higher at 55N when hybrids prior to the 1990s were compared with hybrids after the 1990s in ACRE and PPAC, respectively, whereas HI was 10 and 11% higher for this comparison at 220N. In contrast, NHI levels were stable in hybrids after 2000 at both N rates and locations, except that the 1967 hybrid had a high NHI at 55N at PPAC (Table 7). The N rate by hybrid interaction of NHI at PPAC was due to a greater response to higher N rate in the 1975 and the 2005s hybrids (increasing rate ranged from 0.03 to 0.04 kg kg^−1^) compared with rest of hybrids (increasing rate ranged from 0.00 to 0.02 kg kg^−1^).

As for plant N status, even though higher N rates increased NNI by 0.22 at both locations, PPAC still had an NNI below 1.0 even at the high N rate whereas maize plants at ACRE were only categorized as deficient at low N but not at the high N rate (Table 7). At low N, the NNI was not different among hybrids at either location. As N supply increased, all hybrids reached NNI levels above 1.0 with 220N at ACRE (indicating a possible luxury N uptake occurred in all hybrids, and especially in the 2005 hybrids).

In general, the higher N rate decreased PFP, NIE, and NCE at both locations, and increased HI, NNI at both locations, but increased NHI only at PPAC (Table 7). There were no density and hybrid interactions for PFP, NIE, NCE, HI, NHI, and NNI at either location (Table 7). Density increased PFP at ACRE when density increased from low to medium density, but PFP decreased when density increased from medium to high density. Density had no impact on PFP at PPAC. Higher densities increased NIE (ACRE) and NCE (both locations), but decreased NHI (both locations).

### Dissection of nitrogen internal efficiency

The proportion of NIE variation that was explained by other measurement factors depended on the different explanatory variables examined. To examine the underlying causes of NIE variation, log-transformation was used for both predictor and explanatory variables to obtain linear regression.

**Figure 2:**
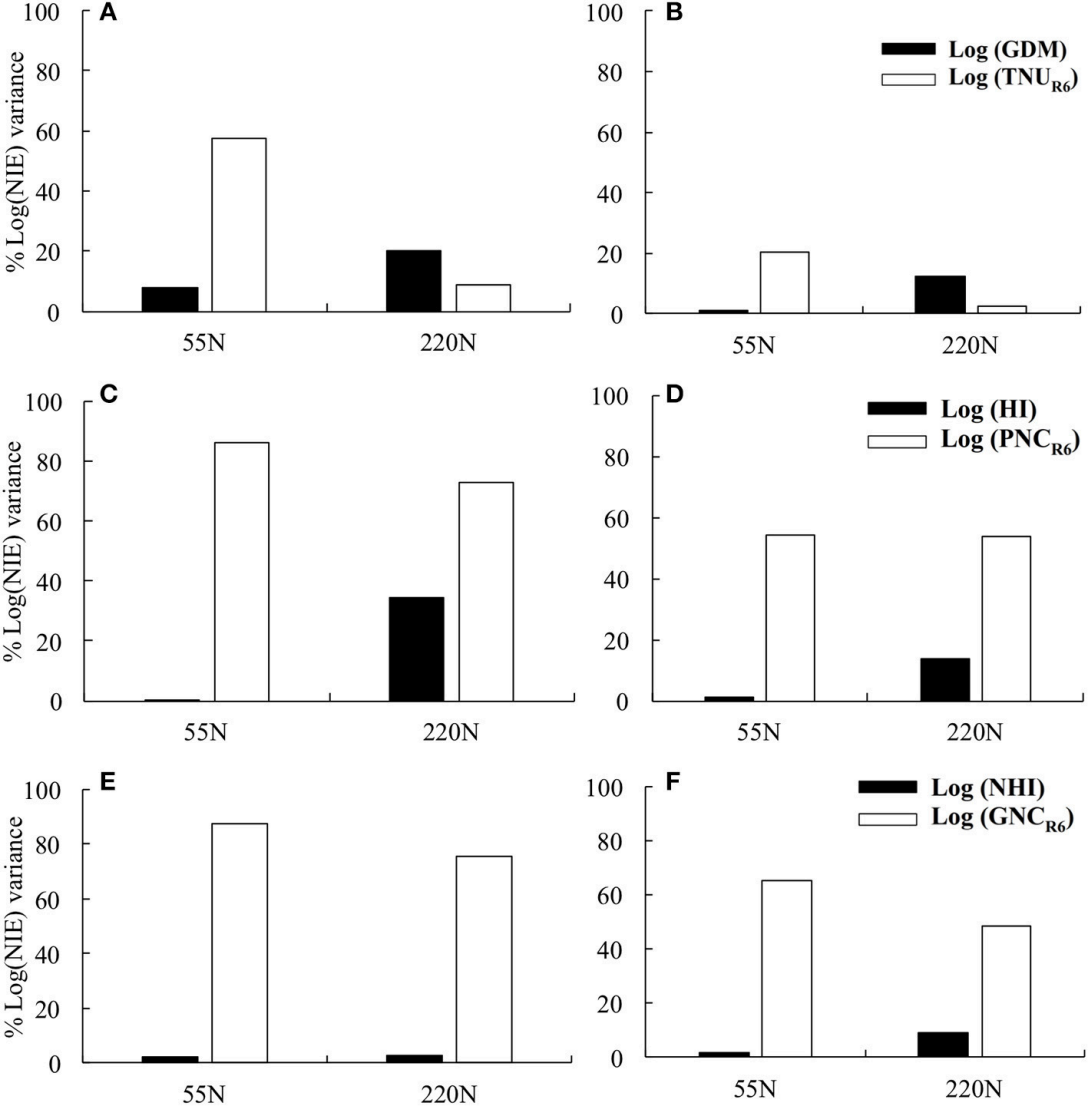
The percentage of Log (NIE) variance explained by log (grain dry matter) and log (total N content at maturity) at 55 and 220N at ACRE **(A)** and PPAC **(B)**; explained by log (HI) and log (plant N concentration) at maturity at 55 and 220N at ACRE **(C)** and PPAC **(D)**; explained by log (NHI) and log (grain N concentration at maturity) at 55 and 220N at ACRE **(E)** and PPAC **(F)**.

**Figure 3:**
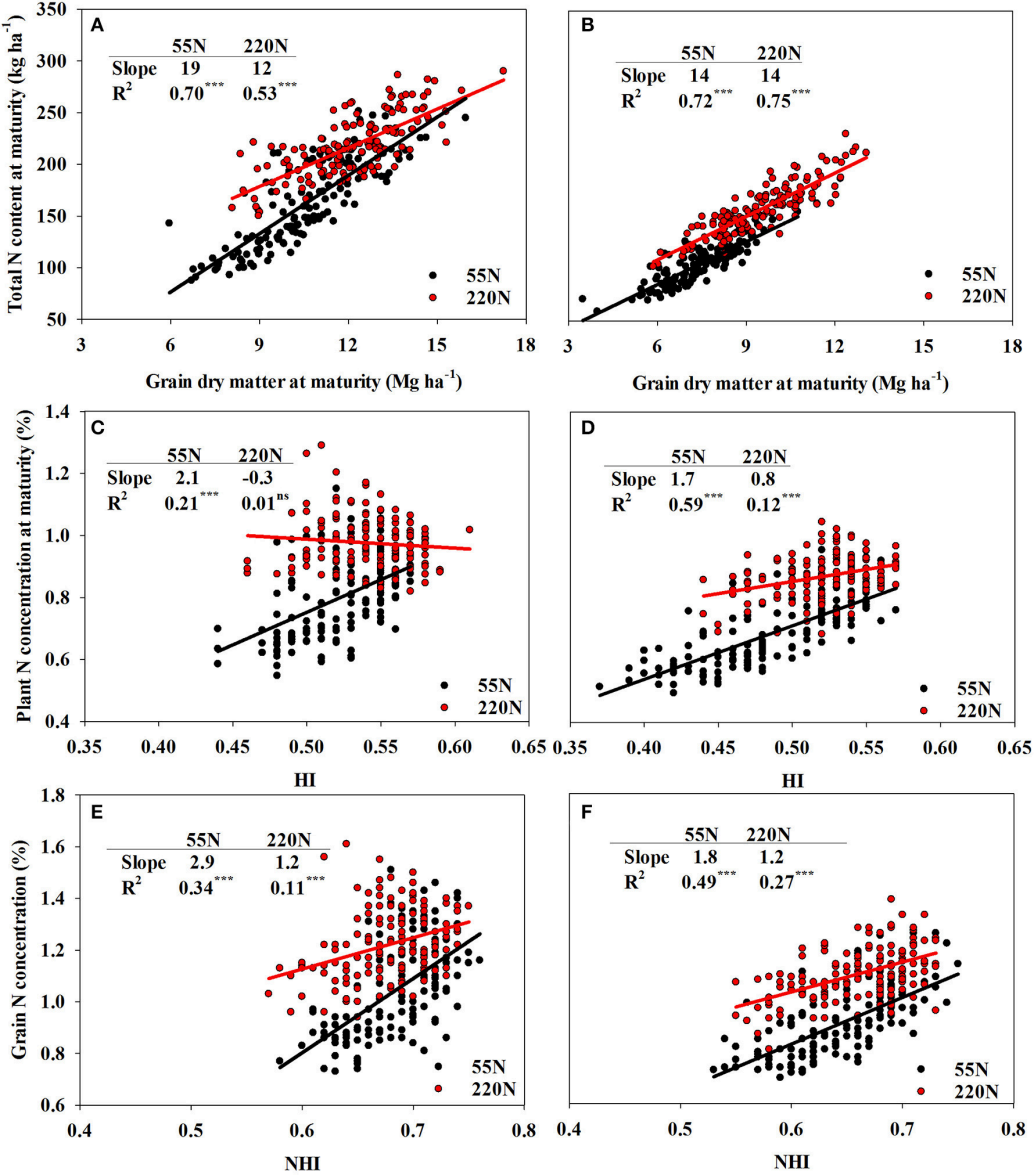
The auto-correlation of total N concentration and grain dry matter at R6 at both N rates at ACRE **(A)** and PPAC **(B)**; plant N concentration and HI at both N rates at ACRE **(C)** and PPAC **(D)**; grain N concentration and NHI at both rates at ACRE **(E)** and PPAC **(F)**. The slope differences between low N and high N rates are: **(A)** 7^***^, **(B)** 0^ns^, **(C)** 2.4^***^, **(D)** 0.9^***^, **(E)** 1.7^**^, and **(F)** 0.6^*^. ^***^p-value < 0.001; ^**^p-value < 0.01; ^*^p-value < 0.05; ns, not significant (*p*-value > 0.05).

Total N content at maturity explained 57 and 20% of NIE variation at ACRE and PPAC, respectively, at 55N whereas GDM explained 8 and 1% of NIE variation at ACRE and PPAC at 55N, respectively (Figures 2A,B). In contrast, at 220N, GDM explained a higher proportion of NIE variation compared to TNU_R6_ at both locations. The GDM accounted for 20% of NIE variation at ACRE and 12% at PPAC, while TNU_R6_ accounted for 9% of NIE variation at ACRE and 2% at PPAC (Figures 2A,B). Grain dry matter was also highly correlated with TNU_R6_ (Figures 3A,B).

However, when NIE was dissected into PNC_R6_ and HI, NIE variations were dominantly explained by PNC_R6_ at both N rates and both locations (Figures 2C,D). At 55N, 86% of total variance of NIE was explained by PNC_R6_ and only 0.3% was explained by HI at ACRE, and the same metrics were 54 vs. 1% at PPAC. At 220N, PNC_R6_ explained 73 and 54% of total variance of NIE, and HI explained 34 and 14% of variance of NIE at ACRE and PPAC, respectively. The GNC explained even more of variation in NIE compared to NHI when NIE was dissected into GNC and NHI. Grain N concentration at maturity accounted for 87 and 65% of the total variance of NIE at 55N, and 75 and 48% of total NIE variance at 220N, respectively, at ACRE and PPAC (Figures 2E,F). In contrast, NHI accounted for only 2% of total variance of NIE at 55N, and for 3 or 9% of total NIE variance at 220N, respectively, at the same two locations (Figures 2E,F). Increased NHI occurred simultaneously with higher GNC, as evident from the positive correlations between these two variables, even though the coefficient of determination decreased from 0.34 to 0.11 at ACRE and from 0.49 to 0.27 at PPAC when N rate increased from 55 to 220N (Figures 3E,F).

## Discussion

### N rate effects on N dynamics and N use efficiency

Lemaire and Gastal (1997) introduced the use of NNI as a determination standard for plant N status. If NNI is larger than 1.0, the actual plant N concentration should be sufficient for achieving maximum biomass and vice versa (Lemaire and Gastal, 1997). In this study, the NNI responses at two locations suggested very different apparent plant N supplies, with silking-stage N deficiencies occurring at both N rates at PPAC but only at 55N at ACRE (Table 7). Increasing N supply had dramatically different impacts on these two locations. For example, mean GDM was increased by 29% at PPAC (2.1 Mg ha^−1^) and by only 14% at ACRE (1.5 Mg ha^−1^) at the higher N rate (Tables 1, 2). Mean PostN increased by 105% at PPAC (21 kg ha^−1^) and 50% at ACRE (23 kg ha^−1^) at the higher N rate (Tables 5, 6). Ciampitti and Vyn (2011) also reported large GDM and PostN gains with 165–330 kg N ha^−1^ vs. 0 kg N ha^−1^ for four 2,000-era hybrids (regardless of their transgenic insect resistance features) using the same 3 densities that we utilized. In our research, the lack of N rate by hybrid interaction in PostDM and PostN at both locations indicated that all era hybrids responded similarly to greater N supply (Tables 1, 2, 5, 6). However, we observed that the two most recent hybrids achieved higher PostN than all other hybrids at ACRE but not at PPAC (Tables 5, 6), which indicated that these recent hybrids recovered more N as availability increased.

Hybrid selection is usually conducted with N sufficient conditions to insure comparisons are near peak grain production levels (Bänziger et al., 1997; Lafitte et al., 1997). This could lead to limitations or large variation in the resulting hybrid performances when soil N conditions are poor (Lafitte et al., 1997; Worku et al., 2007). Several research groups have addressed the need of conducting selection under low N conditions. For example, Moll et al. (1982) showed that conducting hybrid selection under low N was more effective at identifying high NIE genotypes. Worku et al. (2007) reported on the importance of PostN and NIE selection factors in improving grain yield under poor tropical soil conditions.

In our research, beyond the impact of N rate on PostDM and PostN dynamics, the higher N rate decreased PFP by 115 kg kg^−1^, NIE by 11 kg kg^−1^ and NCE by 30 kg kg^−1^ when averaged over the two locations (Table 7). Overall NIE was 69 kg kg^−1^ at 55N across locations, and it decreased to 58 kg kg^−1^ at 220N. This reduction was consistent across all hybrids as no N rate by hybrid interactions occurred at either location (Table 7). The reduction of NIE at higher N rates agreed with Anderson et al. (1985) and Ciampitti and Vyn (2011).

The N rate also affected the correlations between PNC_R6_ and HI, and between GNC and NHI, which were both stronger at 55 than 220N (Figure 3). Ciampitti and Vyn (2013) documented poor correlations between GNC and NHI (*R*^2^ = 0.14) and found that the slope for log (GNC) and log (NHI) did not differ between old era (1940–1990) and new era (1991–2011) hybrid groups regardless of N rates. In our study, the poor correlations at 220N were related to higher PostDM and higher TDM_R6_, which negatively affected GNC and PNC_R6_ and weakened the effects of NHI and HI. In this case, a high N rate input resulted in less dilution of both PNC_R6_ and GNC even though TDM_R6_ and GDM increased dramatically. This finding agreed with Echarte et al. (2013), who also found reduced protein concentrations in newer hybrids in a series of DeKalb hybrids (1965–1993) when comparisons were made under zero N fertilized treatments, but not with N fertilizer applications.

The results of this study demonstrate the tradeoffs of how adding fertilizer N leads to greater DM accumulation by GDM and TDM_R6_ (as well as PostDM and PostN), but leads to reduced PFP, NIE, and NCE. However, these tradeoffs were smaller at the more N deficient location even when the same N fertilizer rate was added. Moreover, the results also indicated that variation of selected traits could be large under severe N deficiency conditions if hybrid selection for selected traits were only conducted in adequate soil N conditions.

### Enhancing nitrogen internal efficiency

There was almost no gain apparent in NIE with more recent hybrids in this study, and what NIE gain there was relative to the oldest 1967 hybrid became fairly stable by the 1994 hybrid (Table 7). The Ciampitti and Vyn (2012) review of historic experiments documented an increase in NIE (49.7–56.0 kg kg^−1^) when older hybrids (1940–1990) were compared with newer hybrids (1991–2011) with the same average N rates in the context of an average increase in plant density from 5.6 to 7.0 plants m^−1^. The gains in NIE observed with plant densities across hybrid eras in this study, as well as for post-2000 hybrids by Ciampitti and Vyn (2011) suggests that conclusions about mean hybrid era impacts on NIE aren't fully valid unless comparisons are made at the same densities. Until more research is conducted, it is also possible to speculate that the grain yield improvements achieved in the Dekalb hybrids for this maturity zone did not coincide with the accompanying NIE gains observed in the maize genetic improvements of other commercial or public breeding programs. Certainly, the morphological changes in relative kernel weight gains observed in these Dekalb hybrids over time was rather unique (Chen et al., 2017).

The reasons for the NIE plateau in more recent hybrids in our research were exploited by dissecting NIE into its components of PNC_R6_ and HI, or GNC and NHI. Plant N concentration at maturity and GNC had a dominant impact on NIE across all treatments (Figure 2). Newer hybrids did not have the lowest PNC_R6_ or GNC across all treatments (Tables 3, 4) despite the larger kernel weights achieved in these newer hybrids (Chen et al., 2017) and the observation by Ciampitti and Vyn (2012) of a mean 10% decrease in both PNC_R6_ and GNC in experiments utilizing hybrids after 1990. In our direct comparison on hybrids side by side, there was no consistent era effect on the proportion of NIE variation explained by either PNC_R6_ or GNC.

**Figure 4:**
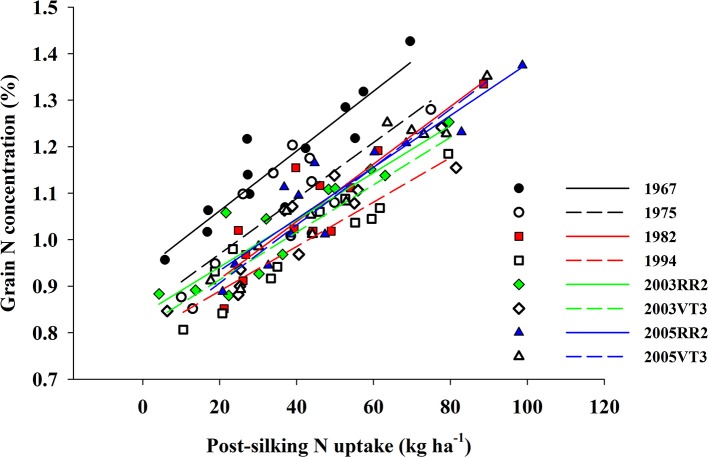
The correlation between grain N concentration (%) and post-silking N uptake (kg ha^−1^) for all eight hybrids when averaged across all treatments and locations. 1967: *GNC* = 0.9 + 0.006 × *PostN, R*^2^ = 0.84, *p* < 0.001; 1975: *GNC* = 0.8 + 0.006 × *PostN, R*^2^ = 0.67, *p* = 0.001; 1982: *GNC* = 0.8 + 0.006 × *PostN, R*^2^ = 0.81, *p* < 0.001; 1994: *GNC* = 0.8 + 0.005 × *PostN, R*^2^ = 0.85, *p* < 0.001; 2003RR2: *GNC* = 0.8 + 0.005 × *PostN, R*^2^ = 0.83, *p* < 0.001; 2003VT3: *GNC* = 0.8 + 0.005 × *PostN, R*^2^ = 0.84, *p* < 0.001; 2005RR2: *GNC* = 0.8 + 0.006 × *PostN, R*^2^ = 0.85, *p* < 0.001; 2005VT3: *GNC* = 0.8 + 0.006 × *PostN, R*^2^ = 0.93, *p* < 0.001.

**Figure 5:**
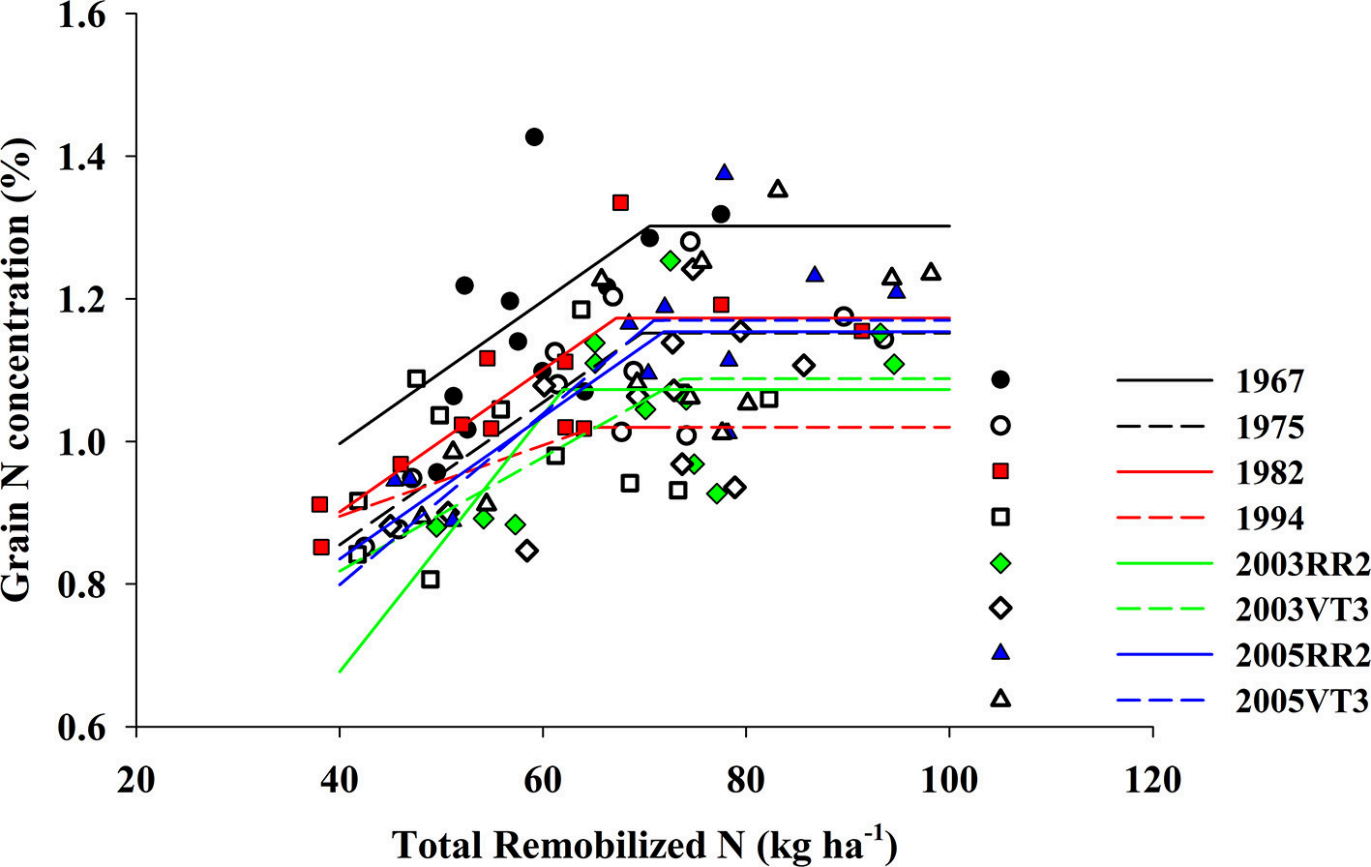
Bilinear model fitted for grain N concentration (%) and total remobilized N (kg ha^−1^) for eight hybrids. Slopes for eight hybrids are (order from 1967 to 2005VT3): 0.010, 0.010, 0.010, 0.005, 0.018, 0.008, 0.010, and 0.012. The threshold (*x*_0_) for eight hybrids are (order from 1967 to 2005VT3): 70, 70, 67, 65, 62, 74, 72, and 71 kg ha^−1^. The plateau for eight hybrids are (order from 1967 to 2005VT3): 1.30, 1.15, 1.17, 1.02, 1.07, 1.09, 1.15 and 1.17. And the *R*^2^ for fitted models are (order from 1967 to 2005VT3): 0.34, 0.65, 0.68, 0.19, 0.49, 0.45, 0.50 and 0.49. *P*-value for all fitted model are less than 0.05.

We observed a strong positive correlation between GNC and PostN, and all hybrids responded similarly (the largest difference among slopes was 0.0015; *p*-value = 0.08) (Figure 4). In contrast, RemN affected GNC in a bilinear way for all hybrids (Figure 5). The lowest plateau for GNC was 1.02 g 100 g^−1^ for the 1994 hybrid and the highest plateau for GNC was 1.30 g 100 g^−1^ for the 1967 hybrid. Other authors have also reported correlations between GNC and PostN or RemN. Coque and Gallais (2007) showed that GNC had higher correlation with PostN than RemN when inbred lines were tested with a common 150 kg N ha^−1^ and 90,000 plants ha^−1^. Anderson et al. (1985) reported that higher N remobilization during grain filling period benefited NIE in prolific hybrids when then-current hybrids were compared. However, in this study, both PostN and RemN had positive effects on GNC which directly resulted in a lower NIE. Moreover, the correlation coefficient with NIE was much higher for PostN (−0.58, *p* < 0.001) compared to RemN (−0.33, *p* < 0.001).

Apparently, in the DeKalb hybrids used here, enhanced NIE occurred inconsistently and only via lower GNC or PNC_R6_, which becomes more and more difficult to achieve as there will be a minimum GNC or PNC_R6_ to maintain seed nutrient levels or grain yields (Ladha et al., 2005; Ciampitti and Vyn, 2013; Gastal et al., 2015). Furthermore, lower GNC or PNC_R6_ may be undesirable for another N efficiency parameter because lower N fertilizer recovery efficiencies (NRE) occur when plant N concentrations at maturity are lowered. Lower NRE following N fertilizer applications will enhance N losses to the atmosphere (Omonode et al., 2017). However, we could not measure NRE in this study because there was no zero N control treatment.

Only very small proportions of NIE variation were explained by either NHI or HI in this study (Figure 2), which appeared to be related to lack of improvement of NHI and HI *per se* across the 38-year period in this hybrid series. The NHI difference between hybrids prior to 2000 and after 2000 only averaged 4% across treatments and locations, and the HI difference between hybrids prior to and after 1994 was only 4% (Table 7). Ciampitti and Vyn (2013) also indicated the lack of incremental gains in NHI and HI as era increased.

In this research, the lack of improvement of HI and NHI was related to lack of apparent era benefits to both the proportion of PostDM to TDM_R6_ and in the proportion of PostN to TNU_R6_, even though both PostDM and PostN *per se* were increasing in more recent hybrids (Tables 1, 2, 5, 6). Gastal et al. (2015) indicated the difficulty of increasing NIE by dilution of grain N concentration because of the lack of variation in N dilution curves between genotypes; they considered that achieving more genotype variation in NHI and HI was a more likely pathway to enhanced NIE. Hence, although higher PostN and PostDM were found in this study, the lack of improvement in the proportions of PostDM to TDM_R6_, and PostN to TNU_R6_, in more recent hybrids accounts for the lack of hybrid era differences in NHI and HI.

## Conclusion

This study focused on understanding N use efficiency changes over 38 years of DeKalb commercial hybrid production in the context of post-silking dry matter and N dynamics associated with plant density and N rate variables. We found that there was no gain in TDM_R1_ after the 1975 hybrid, and that all hybrids from 1975 to 2005 were surprisingly consistent in TDM_R1_ regardless of the plant density or N rates they were compared under. However, at maturity, we found that TDM_R6_ gains averaged 80 kg ha^−1^ year^−1^ across locations, N rates, and plant densities. There was no consistent era effect on the ratio of PostDM to TDM_R6_.

The two most recent hybrids (2005 hybrids) had lowest LNC and SNC at maturity but did not show declines in GNC and PNC_R6_ compared to the 1967 hybrid in this study. Total N contents at R1 and R6 were all higher in the 2003 and 2005 hybrids; overall TNU_R6_ increased 0.68 kg ha^−1^ year^−1^, with a yearly increasing rate of GNU at 0.54 kg N ha^−1^ year^−1^. Both higher PostN and RemN contributed to GNU gains in more recent hybrids. The increases in PostN uptake were more consistent at ACRE with 0.3 kg N ha^−1^ year^−1^. There were no consistent era gains in the ratios of PostN/GNU and PostN/TNU_R6_ at either location.

Partial factor productivity increased 0.9 kg kg^−1^ year^−1^ at the low N rate and 0.3 kg kg^−1^ year^−1^ at high N rate. Hybrid differences in NIE and NCE didn't demonstrate any consistent era effects, but NIE and NCE gains were evident at higher plant densities even though densities didn't impact PFP. The tradeoff between reduction in PFP, NIE and NCE and improvement of PostDM and PostN at higher N rate was smaller at the more severely N deficient location (PPAC) due to a greater increase in PostDM and PostN. Moreover, the lack of NIE gains in this study can be attributed to the more dominant roles of N rate and plant density factors plus the little reduction of GNC and PNC_R6_ noted in progressively newer hybrids. Nevertheless, GNC and PNC_R6_ accounted for most of the NIE variance across treatments and locations.

The lack of improvement in HI (which reached a plateau in the 1990s) and NHI (which reached a plateau in 2003 hybrids) limited their potential contribution to achieving the enhanced NIE in more recent hybrids that was anticipated from the literature. The absence of any era benefit in the proportions of PostDM to TDM_R6_ and of PostN to TNU_R6_ restricted any possible improvements of HI and NHI over this 38-year period of hybrid introductions. This study demonstrated that depending too much on a dilution of GNC and PNC_R6_ over time in hybrid improvement programs would not be a reliable solution for increasing NIE. Instead, enhancing HI and NHI could be of more benefit to achieve further genetic increases in NIE if hybrid era gains in the proportions of PostDM to TDM_R6_ and PostN to TNU_R6_ were realized.

## Author contributions

KC collected and analyzed the maize phenotypic data for her Ph.D. thesis. TV designed the experimental approach. KC and TV co-wrote the manuscript.

### Conflict of interest statement

The authors declare that the research was conducted in the absence of any commercial or financial relationships that could be construed as a potential conflict of interest.
