# Supplementary material for: Post-silking Factor Consequences for N Efficiency Changes Over 38 Years of Commercial Maize Hybrids
Source: Front Plant Sci. 2017 Oct 11;8:1737. doi: 10.3389/fpls.2017.01737 (PMC5641558; doi:10.3389/fpls.2017.01737)
Supplement: Supplementary file 1 [file Table1.DOCX]

Table S1. ANOVA of main effects for leaf, stem and ear dry matter at silking, for leaf, stem, cob dry matter at maturity, and for leaf, stem dry matter remobilization from silking to maturity at ACRE.

|  |  | **Dry matter at silking** | | |  | **Dry matter at silking** | | |  | **Remobilization** | |  |
| --- | --- | --- | --- | --- | --- | --- | --- | --- | --- | --- | --- | --- |
|  |  | **Leaf** | **Stem** | **Ear** |  | **Leaf** | **Stem** | **Cob** |  | **Leaf** | **Stem** | |
|  |  | **Mg ha^-1^** | | |  | **Mg ha^-1^** | | |  | **Mg ha^-1^** | |  |
| **N rate** | **55N** | 2.7 | 6.4 | 0.36 |  | 3.1 | 5.3 | 1.53 |  | -0.31 | 1.1 | |
| **(N)** | **220N** | 2.9 | 6.5 | 0.35 |  | 3.2 | 5.4 | 1.68 |  | -0.31 | 1 | |
|  | ***LSD*** | ***0.1*** | ***0.4*** | ***0.05*** |  | ***0.3*** | ***0.4*** | ***0.15*** |  | 0.37 | ***0.5*** | |
|  |  |  |  |  |  |  |  |  |  |  |  | |
| **Density** | **54,000** | 2.4 | 6 | 0.36 |  | 2.8 | 5.3 | 1.61 |  | -0.42 | 0.7 | |
| **(D)** | **79,000** | 2.8 | 6.5 | 0.38 |  | 3.1 | 5.3 | 1.62 |  | -0.3 | 1.2 | |
|  | **104,000** | 3.1 | 6.8 | 0.33 |  | 3.4 | 5.4 | 1.59 |  | -0.22 | 1.4 | |
|  | ***LSD*** | ***0.1*** | ***0.3*** | ***0.05*** |  | ***0.1*** | ***0.3*** | ***0.05*** |  | 0.14 | ***0.4*** | |
|  |  |  |  |  |  |  |  |  |  |  |  | |
| **Hybrid** | **1967** | 2.2 | 5.2 | 0.51 |  | 2.5 | 4.1 | 1.58 |  | -0.36 | 1.1 | |
| **(H)** | **1975** | 2.9 | 6.8 | 0.15 |  | 3.3 | 5.7 | 1.58 |  | -0.42 | 1.1 | |
|  | **1982** | 3 | 6.7 | 0.17 |  | 3.5 | 5.9 | 1.59 |  | -0.51 | 0.8 | |
|  | **1994** | 2.7 | 6.5 | 0.38 |  | 2.8 | 5.3 | 1.45 |  | -0.04 | 1.2 | |
|  | **2003RR2** | 2.9 | 6.5 | 0.38 |  | 2.9 | 5.3 | 1.67 |  | -0.07 | 1.3 | |
|  | **2003VT3** | 2.7 | 6.6 | 0.38 |  | 2.9 | 5.2 | 1.57 |  | -0.2 | 1.4 | |
|  | **2005RR2** | 3 | 6.6 | 0.46 |  | 3.5 | 5.8 | 1.67 |  | -0.48 | 0.9 | |
|  | **2005VT3** | 3 | 6.4 | 0.42 |  | 3.4 | 5.5 | 1.67 |  | -0.43 | 1 | |
|  | ***LSD*** | ***0.1*** | ***0.3*** | ***0.07*** |  | ***0.2*** | ***0.3*** | ***0.07*** |  | 0.19 | ***0.4*** | |
|  |  |  |  |  |  |  |  |  |  |  |  | |
| **F-test** | **N** | 0.043 | ns | ns |  | ns | ns | ns |  | ns | ns | |
|  | **D** | <0.001 | <0.001 | ns |  | <0.001 | ns | ns |  | 0.024 | 0.011 | |
|  | **H** | <0.001 | <0.001 | <0.001 |  | <0.001 | <0.001 | <.0001 |  | <0.001 | 0.039 | |
|  | **N × D** | ns | ns | ns |  | ns | ns | ns |  | ns | ns | |
|  | **N × H** | ns | ns | ns |  | ns | 0.032 | ns |  | ns | ns | |
|  | **D × H** | ns | ns | ns |  | ns | ns | ns |  | ns | ns | |
|  | **N × D× H** | ns | ns | ns |  | ns | ns | ns |  | ns | ns | |
