# Supplementary material for: Post-silking Factor Consequences for N Efficiency Changes Over 38 Years of Commercial Maize Hybrids
Source: Front Plant Sci. 2017 Oct 11;8:1737. doi: 10.3389/fpls.2017.01737 (PMC5641558; doi:10.3389/fpls.2017.01737)
Supplement: Supplementary file 2 [file Table2.DOCX]

Table S2. ANOVA of main effects of leaf, stem, and ear dry matter at silking, for leaf, stem, and cob dry matter at ma
for leaf and stem dry matter remobilization from silking to maturity at PPAC.

		Dry matter at silking			Dry matter at maturity			Remobili
		**Leaf**	**Stem**	**Ear**	**Leaf**	**Stem**	**Cob**	**Leaf**
		Mg ha^{-1}			Mg ha^{-1}			Mg h
**N rate**	**55N**	2.5	6.1	0.21	2.5	4.1	1.23	-0.01
**(N)**	**220N**	2.6	6.2	0.23	2.7	4.5	1.47	-0.17
	LSD	***0.1***	***0.3***	***0.04***	***0.2***	***0.2***	***0.14***	***0.18***
**Density**	**54,000**	2.2	5.8	0.25	2.4	4.2	1.38	-0.18
**(D)**	**79,000**	2.6	6.2	0.21	2.6	4.4	1.38	-0.06
	**104,000**	2.8	6.5	0.19	2.8	4.3	1.28	-0.02
	LSD	***0.1***	***0.2***	***0.03***	***0.2***	***0.1***	***0.03***	***0.15***
**Hybrid**	**1967**	2.1	5.5	0.45	1.9	3.5	1.39	0.14
**(H)**	**1975**	2.6	6.5	0.07	2.6	4.7	1.41	0.01
	**1982**	2.6	6.2	0.07	2.9	4.9	1.31	-0.23
	**1994**	2.3	6.2	0.24	2.5	4.1	1.24	-0.13
	**2003RR2**	2.5	6.2	0.23	2.6	4.2	1.35	-0.03
	**2003VT3**	2.5	6.4	0.23	2.7	4.4	1.36	-0.16
	**2005RR2**	2.7	6.1	0.21	2.8	4.3	1.33	-0.11
	**2005VT3**	2.7	6.2	0.24	2.9	4.3	1.38	-0.18
	LSD	***0.1***	***0.2***	***0.04***	***0.1***	***0.2***	***0.04***	***0.18***
**F-test**	**N**	ns	ns	ns	0.014	0.003	0.006	ns
	**D**	<0.001	<0.001	<0.001	<0.001	0.05	<0.001	ns
	**H**	<0.001	<0.001	<0.001	<0.001	<0.001	<0.001	<0.001
	**N × D**	ns	ns	ns	ns	ns	ns	ns
